# Supplementary material for: Stromal depletion by TALEN-edited universal hypoimmunogenic FAP-CAR T cells enables infiltration and anti-tumor cytotoxicity of tumor antigen-targeted CAR-T immunotherapy
Source: Front Immunol. 2023 May 12;14:1172681. doi: 10.3389/fimmu.2023.1172681 (PMC10213512; doi:10.3389/fimmu.2023.1172681)
Supplement: Supplementary file 1 [file DataSheet_1.docx]

Supplementary Material

**Stromal depletion by TALEN-edited universal hypoimmunogenic FAP-CAR T cells enables infiltration and anti-tumor cytotoxicity of tumor antigen-targeted CAR-T immunotherapy.**

Shipra Das^*^, Julien Valton, Philippe Duchateau, Laurent Poirot

*** Correspondence: Shipra Das: Shipra.das@cellectis.com**

## Supplementary Figures


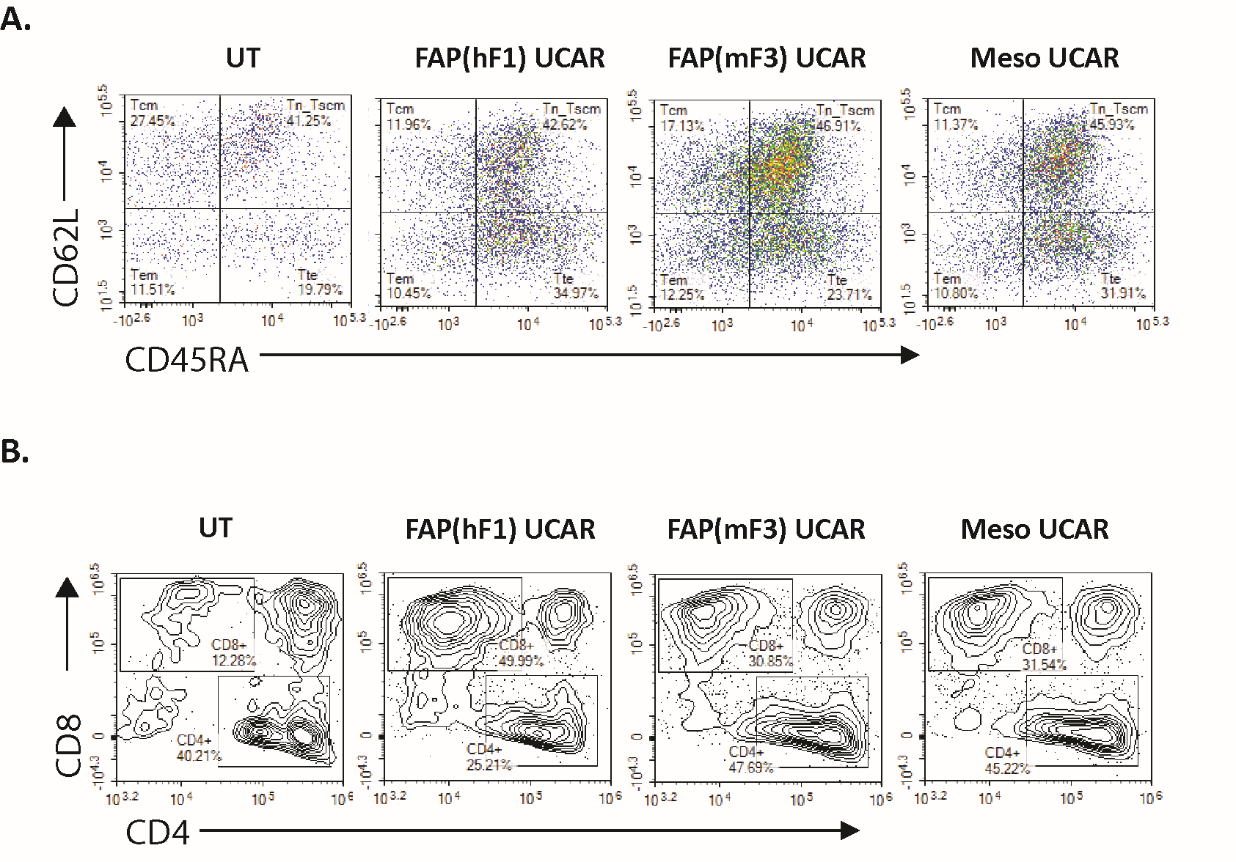


**Figure S1. (A)** Flow cytometry plots of T cell differentiated states of UCAR T-cells as indicated, pre-gated on viable, CAR^+^ singlet cells or on viable, singlet cells for UT control. **(B)** Flow cytometry plots of CD4^+^ and CD8^+^ T-cell sub-populations of UCAR T-cells as indicated, pre-gated on viable, CAR^+^ singlet cells or on viable, singlet cells for UT control.


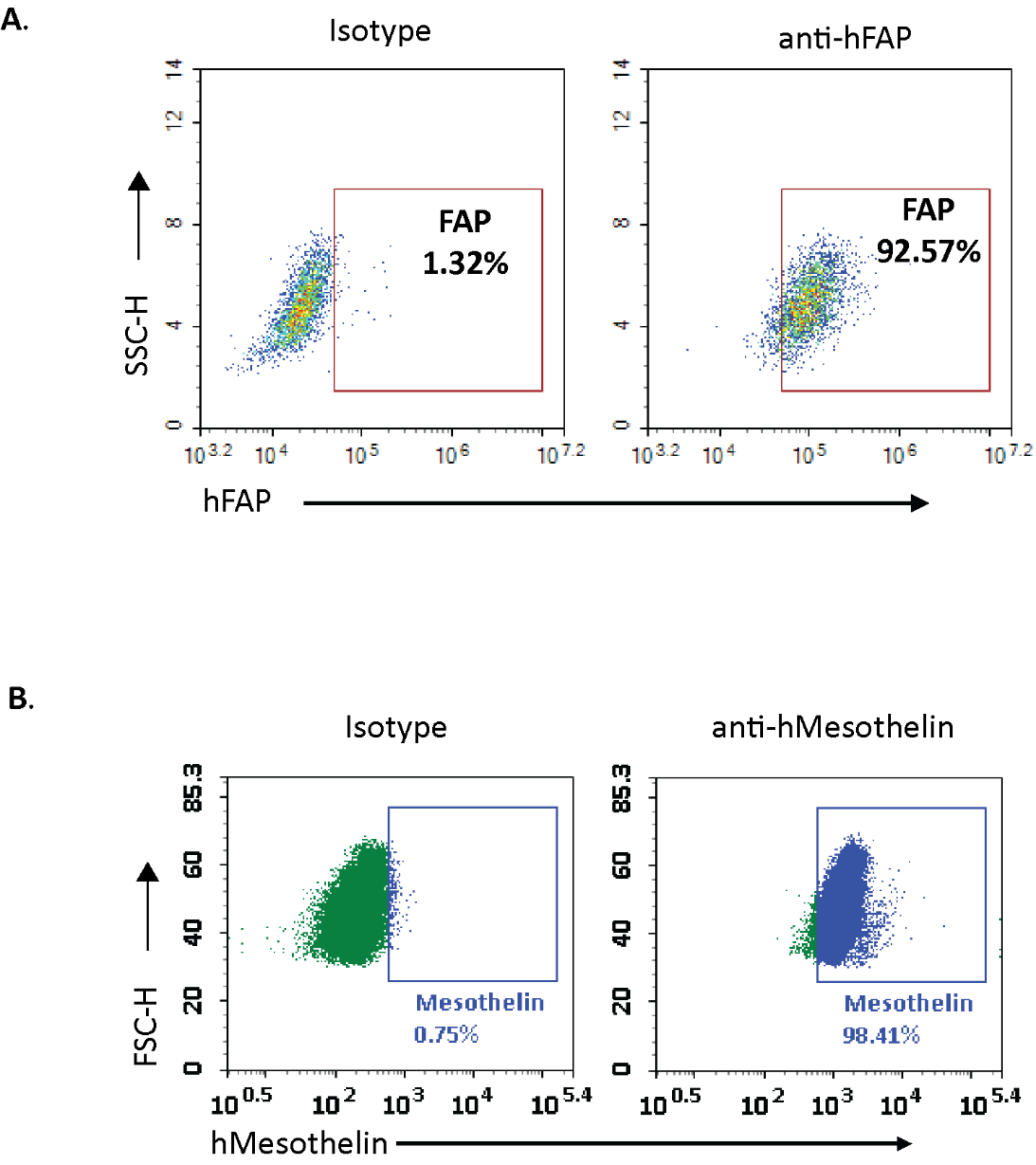


**Figure S2. (A)** Flow cytometry plots depicting staining of patient TNBC-derived CAF cells with isotype control or anti-human FAP protein antibody. **(B)**  Flow cytometry plots depicting staining of TNBC cell line HCC70-GFP with isotype control or anti- human Mesothelin protein antibody.


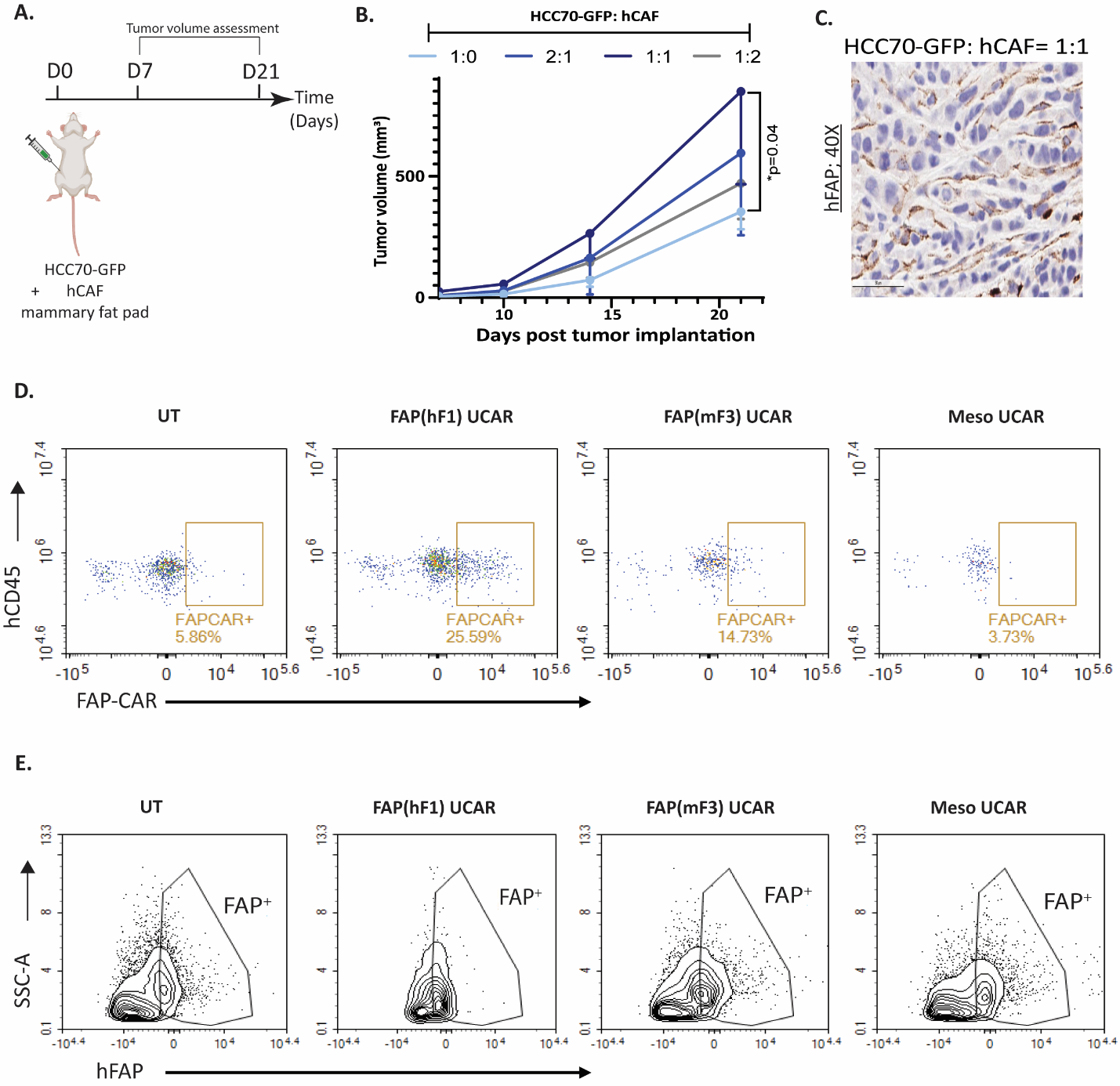


**Figure S3. (A)** Schematic depicting assessment of tumor growth kinetics upon co-implantation of 7 x 10^6^ HCC70-GFP cells with CAFs at different ratios in mammary fat pad of NSG mice. **(B)** Graph depicts growth kinetics of orthotopic mammary tumors implanted as outlined in **(A).** P-value calculated using two-tailed, unpaired student t-test, n=3 per cohort,). **(C)** Immunohistochemical staining of human FAP protein in orthotopic tumor section derived from co-implantation of 7 x 10^6^ HCC70-GFP and 7 x 10^6^ TNBC-derived CAFs in mammary fat pad of NSG mice. **(D)** Flow cytometry plots depicting FAPCAR^+^ cells pre-gated on viable, mouse CD45^-^, human CD45^+^ singlet cells in spleen of orthotopic tumor bearing mice, treated as indicated. **(E)** Flow cytometry plots depicting human FAP^+^ cells pre-gated on viable, mouse CD45^-^, human CD45^-^ singlet cells in orthotopic tumors of NSG mice, treated as indicated.


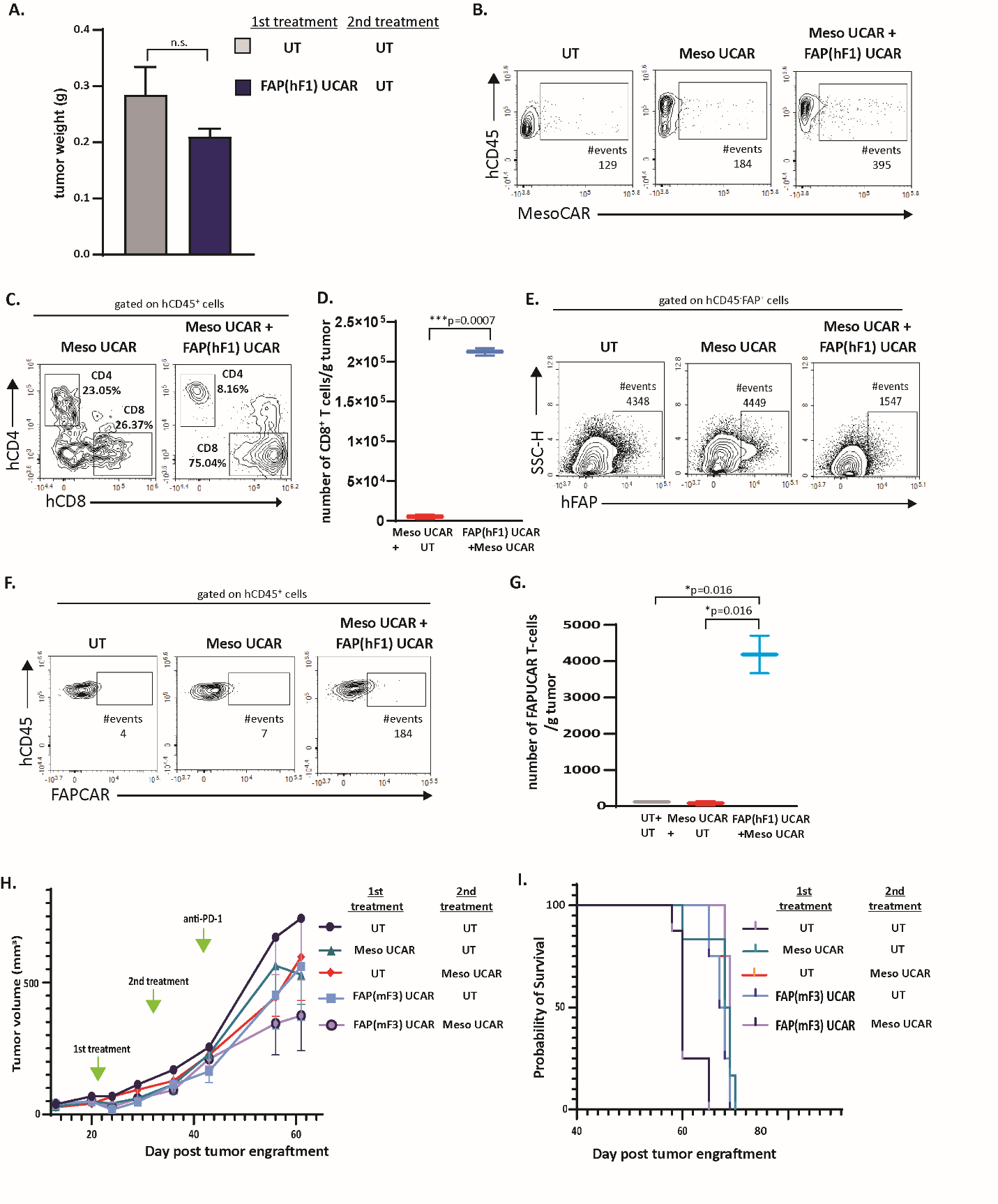


**Figure S4. (A)** Bar graph depicting tumor weight harvested from orthotopically injected NSG mice and treated with UCAR T-cells as indicated, 21 days post treatment initiation. n.s.-not significant. **(B)**  Flow cytometry plots depicting Mesothelin CAR^+^ cells pre-gated on viable, mouse CD45^-^, human CD45^+^ singlet cells in orthotopic tumors of NSG mice, treated as indicated. **(C)** Flow cytometry plots depicting CD4^+^ and CD8^+^ cells pre-gated on viable, mouse CD45^-^, human CD45^+^ singlet cells in orthotopic tumors of NSG mice, treated as indicated. **(D)** Box and whiskers plot representing quantitation of total number of CD8^+^ T-cells per gram orthotopic TNBC tumors from mice treated with indicated UCAR T-cells, as determined by flow cytometry. P-values determined by Student *t* test (two-tailed, unpaired), n=2. **(E)** Flow cytometry plots depicting human FAP^+^ cells pre-gated on viable, mouse CD45^-^, human CD45^-^ singlet cells in orthotopic tumors of NSG mice, treated as indicated. FAP^+^ gate was set using CD45^+^ cells which are negative for FAP. **(F)** Flow cytometry plots depicting FAP CAR^+^ cells pre-gated on viable, mouse CD45^-^, human CD45^+^ singlet cells in orthotopic tumors of NSG mice, treated as indicated. **(G)** Box and whiskers plot representing quantitation of total number of FAP UCAR T-cells per gram orthotopic TNBC tumors from mice treated with indicated UCAR T-cells, as determined by flow cytometry. P-values determined by Student *t* test (two-tailed, unpaired), n=2. **(H)** Graph representing growth kinetics of orthotopic TNBC tumors in mice treated as indicated over time, n=5-8 mice per cohort. **(I)** Kaplan–Meier curve for survival analysis of orthotopic TNBC tumor-implanted NSG mice treated as indicated (n=5-8 per cohort).


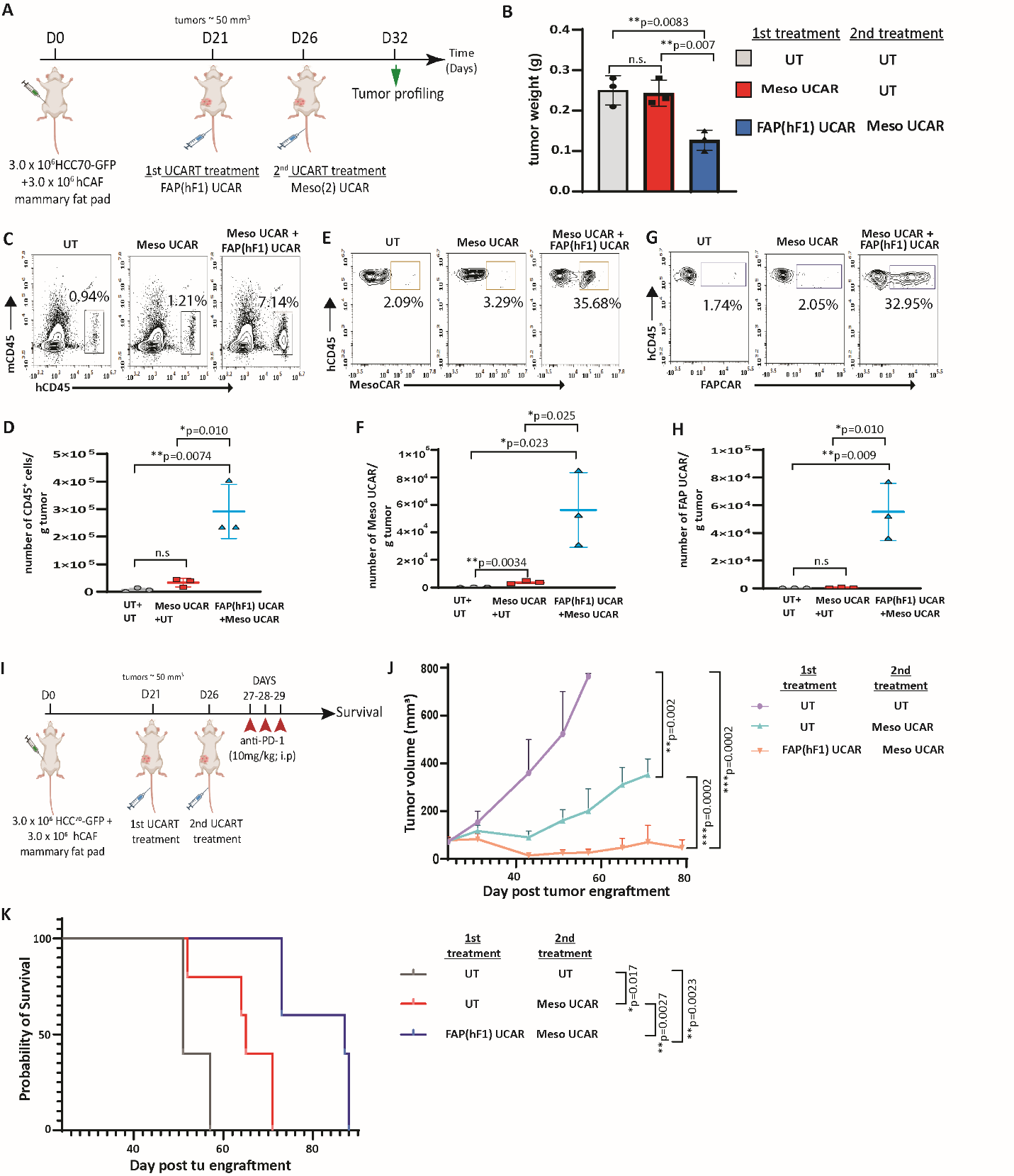


**Figure S5. (A)** Schematic of sequential UCAR T-cell treatment and analysis of orthotopic TNBC tumors implanted in NSG mice. **(B)** Bar graph depicting tumor weight harvested from orthotopically injected NSG mice and treated with UCAR T-cells as indicated, 11 days post treatment initiation. n.s.-not significant. **(C)** Flow cytometry plot of human CD45^+^ UCAR T-cell population in orthotopic TNBC tumors harvested from mice treated as indicated. **(D)** Scatter plot depicting mean and s.d., representing quantitation of total number of CD45^+^ cells per gram tumor, calculated using **(C).** P-values determined by Student *t* test (two-tailed, unpaired), n=3. **(E)** Flow cytometry plots depicting Mesothelin CAR^+^ cells pre-gated on viable, mouse CD45^-^, human CD45^+^ singlet cells in orthotopic tumors of NSG mice, treated as indicated. **(F)** Scatter plot depicting mean and s.d., representing quantitation of total number of Mesothelin CAR^+^ cells per gram tumor, calculated using **(E).** P-values determined by Student *t* test (two-tailed, unpaired), n=3. **(G)** Flow cytometry plots depicting FAP CAR^+^ cells pre-gated on viable, mouse CD45^-^, human CD45^+^ singlet cells in orthotopic tumors of NSG mice, treated as indicated. **(H)** Scatter plot depicting mean and s.d., representing quantitation of total number of FAP CAR^+^ cells per gram tumor, calculated using **(G).** P-values determined by Student *t* test (two-tailed, unpaired), n=3. **(I)** Schematic of sequential UCAR T-cell and anti-PD-1checkpoint inhibitor treatment and subsequent analysis of orthotopic TNBC tumor-implanted NSG mice. **(J)** Graph representing growth kinetics of orthotopic TNBC tumors in mice treated as indicated over time, n=5 mice per cohort. P-values determined by Student *t* test (two-tailed, unpaired). **(K)** Kaplan–Meier curve for survival analysis of orthotopic TNBC tumor-implanted NSG mice treated as indicated (n=5 per cohort). P-values determined by Log-Rank (Mantel-Cox) test.

**Supplemental Tables**

**Supplementary Table S1.** DNA sequences of rlv-CARs

**Supplementary Table S2.** TALEN RNA sequence

| TRAC TALEN LEFT arm | AUGGGCGAUCCUAAAAAGAAACGUAAGGUCAUCGAUAUCGCCGAUCUACGCACGCUCGGCUACAGCCAGCAGCAACAGGAGAAGAUCAAACCGAAGGUUCGUUCGACAGUGGCGCAGCACCACGAGGCACUGGUCGGCCACGGGUUUACACACGCGCACAUCGUUGCGUUAAGCCAACACCCGGCAGCGUUAGGGACCGUCGCUGUCAAGUAUCAGGACAUGAUCGCAGCGUUGCCAGAGGCGACACACGAAGCGAUCGUUGGCGUCGGCAAACAGUGGUCCGGCGCACGCGCUCUGGAGGCCUUGCUCACGGUGGCGGGAGAGUUGAGAGGUCCACCGUUACAGUUGGACACAGGCCAACUUCUCAAGAUUGCAAAACGUGGCGGCGUGACCGCAGUGGAGGCAGUGCAUGCAUGGCGCAAUGCACUGACGGGUGCCCCGCUCAACUUGACCCCCCAGCAGGUGGUGGCCAUCGCCAGCAAUGGCGGUGGCAAGCAGGCGCUGGAGACGGUCCAGCGGCUGUUGCCGGUGCUGUGCCAGGCCCACGGCUUGACCCCCCAGCAGGUGGUGGCCAUCGCCAGCAAUAAUGGUGGCAAGCAGGCGCUGGAGACGGUCCAGCGGCUGUUGCCGGUGCUGUGCCAGGCCCACGGCUUGACCCCCCAGCAGGUGGUGGCCAUCGCCAGCAAUGGCGGUGGCAAGCAGGCGCUGGAGACGGUCCAGCGGCUGUUGCCGGUGCUGUGCCAGGCCCACGGCUUGACCCCGGAGCAGGUGGUGGCCAUCGCCAGCCACGAUGGCGGCAAGCAGGCGCUGGAGACGGUCCAGCGGCUGUUGCCGGUGCUGUGCCAGGCCCACGGCUUGACCCCGGAGCAGGUGGUGGCCAUCGCCAGCCACGAUGGCGGCAAGCAGGCGCUGGAGACGGUCCAGCGGCUGUUGCCGGUGCUGUGCCAGGCCCACGGCUUGACCCCGGAGCAGGUGGUGGCCAUCGCCAGCCACGAUGGCGGCAAGCAGGCGCUGGAGACGGUCCAGCGGCUGUUGCCGGUGCUGUGCCAGGCCCACGGCUUGACCCCGGAGCAGGUGGUGGCCAUCGCCAGCAAUAUUGGUGGCAAGCAGGCGCUGGAGACGGUGCAGGCGCUGUUGCCGGUGCUGUGCCAGGCCCACGGCUUGACCCCGGAGCAGGUGGUGGCCAUCGCCAGCCACGAUGGCGGCAAGCAGGCGCUGGAGACGGUCCAGCGGCUGUUGCCGGUGCUGUGCCAGGCCCACGGCUUGACCCCGGAGCAGGUGGUGGCCAUCGCCAGCAAUAUUGGUGGCAAGCAGGCGCUGGAGACGGUGCAGGCGCUGUUGCCGGUGCUGUGCCAGGCCCACGGCUUGACCCCCCAGCAGGUGGUGGCCAUCGCCAGCAAUAAUGGUGGCAAGCAGGCGCUGGAGACGGUCCAGCGGCUGUUGCCGGUGCUGUGCCAGGCCCACGGCUUGACCCCGGAGCAGGUGGUGGCCAUCGCCAGCAAUAUUGGUGGCAAGCAGGCGCUGGAGACGGUGCAGGCGCUGUUGCCGGUGCUGUGCCAGGCCCACGGCUUGACCCCCCAGCAGGUGGUGGCCAUCGCCAGCAAUGGCGGUGGCAAGCAGGCGCUGGAGACGGUCCAGCGGCUGUUGCCGGUGCUGUGCCAGGCCCACGGCUUGACCCCGGAGCAGGUGGUGGCCAUCGCCAGCAAUAUUGGUGGCAAGCAGGCGCUGGAGACGGUGCAGGCGCUGUUGCCGGUGCUGUGCCAGGCCCACGGCUUGACCCCCCAGCAGGUGGUGGCCAUCGCCAGCAAUGGCGGUGGCAAGCAGGCGCUGGAGACGGUCCAGCGGCUGUUGCCGGUGCUGUGCCAGGCCCACGGCUUGACCCCGGAGCAGGUGGUGGCCAUCGCCAGCCACGAUGGCGGCAAGCAGGCGCUGGAGACGGUCCAGCGGCUGUUGCCGGUGCUGUGCCAGGCCCACGGCUUGACCCCUCAGCAGGUGGUGGCCAUCGCCAGCAAUGGCGGCGGCAGGCCGGCGCUGGAGAGCAUUGUUGCCCAGUUAUCUCGCCCUGAUCCGGCGUUGGCCGCGUUGACCAACGACCACCUCGUCGCCUUGGCCUGCCUCGGCGGGCGUCCUGCGCUGGAUGCAGUGAAAAAGGGAUUGGGGGAUCCUAUCAGCCGUUCCCAGCUGGUGAAGUCCGAGCUGGAGGAGAAGAAAUCCGAGUUGAGGCACAAGCUGAAGUACGUGCCCCACGAGUACAUCGAGCUGAUCGAGAUCGCCCGGAACAGCACCCAGGACCGUAUCCUGGAGAUGAAGGUGAUGGAGUUCUUCAUGAAGGUGUACGGCUACAGGGGCAAGCACCUGGGCGGCUCCAGGAAGCCCGACGGCGCCAUCUACACCGUGGGCUCCCCCAUCGACUACGGCGUGAUCGUGGACACCAAGGCCUACUCCGGCGGCUACAACCUGCCCAUCGGCCAGGCCGACGAAAUGCAGAGGUACGUGGAGGAGAACCAGACCAGGAACAAGCACAUCAACCCCAACGAGUGGUGGAAGGUGUACCCCUCCAGCGUGACCGAGUUCAAGUUCCUGUUCGUGUCCGGCCACUUCAAGGGCAACUACAAGGCCCAGCUGACCAGGCUGAACCACAUCACCAACUGCAACGGCGCCGUGCUGUCCGUGGAGGAGCUCCUGAUCGGCGGCGAGAUGAUCAAGGCCGGCACCCUGACCCUGGAGGAGGUGAGGAGGAAGUUCAACAACGGCGAGAUCAACUUCGCGGCCGACUGAUAACUCGAGGCUGCCUUCUGCGGGGCUUGCCUUCUGGCCAUGCCCUUCUUCUCUCCCUUGCACCUGUACCUCUUGGUCUUUGAAUAAAGCCUGAGUAGGAAGGUCGAGGCGGCCAACAACAAAAAAAAAAAAAAAAAAAAAAAAAAAAAAAAAAAAAAAAAAAAAAAAAAAAAAAAAAAAAAAAAAAAAAAAAAAAAAAAAAAAAAAAAAAAAAAAAAAAAAAAAAAAAAAAAAAAAAAAA |
| --- | --- |
| TRAC TALEN RIGHT arm | AUGGGCGAUCCUAAAAAGAAACGUAAGGUCAUCGAUAUCGCCGAUCUACGCACGCUCGGCUACAGCCAGCAGCAACAGGAGAAGAUCAAACCGAAGGUUCGUUCGACAGUGGCGCAGCACCACGAGGCACUGGUCGGCCACGGGUUUACACACGCGCACAUCGUUGCGUUAAGCCAACACCCGGCAGCGUUAGGGACCGUCGCUGUCAAGUAUCAGGACAUGAUCGCAGCGUUGCCAGAGGCGACACACGAAGCGAUCGUUGGCGUCGGCAAACAGUGGUCCGGCGCACGCGCUCUGGAGGCCUUGCUCACGGUGGCGGGAGAGUUGAGAGGUCCACCGUUACAGUUGGACACAGGCCAACUUCUCAAGAUUGCAAAACGUGGCGGCGUGACCGCAGUGGAGGCAGUGCAUGCAUGGCGCAAUGCACUGACGGGUGCCCCGCUCAACUUGACCCCGGAGCAGGUGGUGGCCAUCGCCAGCCACGAUGGCGGCAAGCAGGCGCUGGAGACGGUCCAGCGGCUGUUGCCGGUGCUGUGCCAGGCCCACGGCUUGACCCCCCAGCAGGUGGUGGCCAUCGCCAGCAAUGGCGGUGGCAAGCAGGCGCUGGAGACGGUCCAGCGGCUGUUGCCGGUGCUGUGCCAGGCCCACGGCUUGACCCCGGAGCAGGUGGUGGCCAUCGCCAGCCACGAUGGCGGCAAGCAGGCGCUGGAGACGGUCCAGCGGCUGUUGCCGGUGCUGUGCCAGGCCCACGGCUUGACCCCGGAGCAGGUGGUGGCCAUCGCCAGCAAUAUUGGUGGCAAGCAGGCGCUGGAGACGGUGCAGGCGCUGUUGCCGGUGCUGUGCCAGGCCCACGGCUUGACCCCCCAGCAGGUGGUGGCCAUCGCCAGCAAUAAUGGUGGCAAGCAGGCGCUGGAGACGGUCCAGCGGCUGUUGCCGGUGCUGUGCCAGGCCCACGGCUUGACCCCGGAGCAGGUGGUGGCCAUCGCCAGCCACGAUGGCGGCAAGCAGGCGCUGGAGACGGUCCAGCGGCUGUUGCCGGUGCUGUGCCAGGCCCACGGCUUGACCCCCCAGCAGGUGGUGGCCAUCGCCAGCAAUGGCGGUGGCAAGCAGGCGCUGGAGACGGUCCAGCGGCUGUUGCCGGUGCUGUGCCAGGCCCACGGCUUGACCCCCCAGCAGGUGGUGGCCAUCGCCAGCAAUAAUGGUGGCAAGCAGGCGCUGGAGACGGUCCAGCGGCUGUUGCCGGUGCUGUGCCAGGCCCACGGCUUGACCCCCCAGCAGGUGGUGGCCAUCGCCAGCAAUAAUGGUGGCAAGCAGGCGCUGGAGACGGUCCAGCGGCUGUUGCCGGUGCUGUGCCAGGCCCACGGCUUGACCCCCCAGCAGGUGGUGGCCAUCGCCAGCAAUGGCGGUGGCAAGCAGGCGCUGGAGACGGUCCAGCGGCUGUUGCCGGUGCUGUGCCAGGCCCACGGCUUGACCCCGGAGCAGGUGGUGGCCAUCGCCAGCAAUAUUGGUGGCAAGCAGGCGCUGGAGACGGUGCAGGCGCUGUUGCCGGUGCUGUGCCAGGCCCACGGCUUGACCCCGGAGCAGGUGGUGGCCAUCGCCAGCCACGAUGGCGGCAAGCAGGCGCUGGAGACGGUCCAGCGGCUGUUGCCGGUGCUGUGCCAGGCCCACGGCUUGACCCCGGAGCAGGUGGUGGCCAUCGCCAGCAAUAUUGGUGGCAAGCAGGCGCUGGAGACGGUGCAGGCGCUGUUGCCGGUGCUGUGCCAGGCCCACGGCUUGACCCCGGAGCAGGUGGUGGCCAUCGCCAGCCACGAUGGCGGCAAGCAGGCGCUGGAGACGGUCCAGCGGCUGUUGCCGGUGCUGUGCCAGGCCCACGGCUUGACCCCCCAGCAGGUGGUGGCCAUCGCCAGCAAUAAUGGUGGCAAGCAGGCGCUGGAGACGGUCCAGCGGCUGUUGCCGGUGCUGUGCCAGGCCCACGGCUUGACCCCUCAGCAGGUGGUGGCCAUCGCCAGCAAUGGCGGCGGCAGGCCGGCGCUGGAGAGCAUUGUUGCCCAGUUAUCUCGCCCUGAUCCGGCGUUGGCCGCGUUGACCAACGACCACCUCGUCGCCUUGGCCUGCCUCGGCGGGCGUCCUGCGCUGGAUGCAGUGAAAAAGGGAUUGGGGGAUCCUAUCAGCCGUUCCCAGCUGGUGAAGUCCGAGCUGGAGGAGAAGAAAUCCGAGUUGAGGCACAAGCUGAAGUACGUGCCCCACGAGUACAUCGAGCUGAUCGAGAUCGCCCGGAACAGCACCCAGGACCGUAUCCUGGAGAUGAAGGUGAUGGAGUUCUUCAUGAAGGUGUACGGCUACAGGGGCAAGCACCUGGGCGGCUCCAGGAAGCCCGACGGCGCCAUCUACACCGUGGGCUCCCCCAUCGACUACGGCGUGAUCGUGGACACCAAGGCCUACUCCGGCGGCUACAACCUGCCCAUCGGCCAGGCCGACGAAAUGCAGAGGUACGUGGAGGAGAACCAGACCAGGAACAAGCACAUCAACCCCAACGAGUGGUGGAAGGUGUACCCCUCCAGCGUGACCGAGUUCAAGUUCCUGUUCGUGUCCGGCCACUUCAAGGGCAACUACAAGGCCCAGCUGACCAGGCUGAACCACAUCACCAACUGCAACGGCGCCGUGCUGUCCGUGGAGGAGCUCCUGAUCGGCGGCGAGAUGAUCAAGGCCGGCACCCUGACCCUGGAGGAGGUGAGGAGGAAGUUCAACAACGGCGAGAUCAACUUCGCGGCCGACUGAUAACUCGAGGCUGCCUUCUGCGGGGCUUGCCUUCUGGCCAUGCCCUUCUUCUCUCCCUUGCACCUGUACCUCUUGGUCUUUGAAUAAAGCCUGAGUAGGAAGGUCGAGGCGGCCAACAACAAAAAAAAAAAAAAAAAAAAAAAAAAAAAAAAAAAAAAAAAAAAAAAAAAAAAAAAAAAAAAAAAAAAAAAAAAAAAAAAAAAAAAAAAAAAAAAAAAAAAAAAAAAAAAAAAAAAAAAAA |
| B2M TALEN LEFT arm | AUGGGCGAUCCUAAAAAGAAACGUAAGGUCAUCGAUAUCGCCGAUCUACGCACGCUCGGCUACAGCCAGCAGCAACAGGAGAAGAUCAAACCGAAGGUUCGUUCGACAGUGGCGCAGCACCACGAGGCACUGGUCGGCCACGGGUUUACACACGCGCACAUCGUUGCGUUAAGCCAACACCCGGCAGCGUUAGGGACCGUCGCUGUCAAGUAUCAGGACAUGAUCGCAGCGUUGCCAGAGGCGACACACGAAGCGAUCGUUGGCGUCGGCAAACAGUGGUCCGGCGCACGCGCUCUGGAGGCCUUGCUCACGGUGGCGGGAGAGUUGAGAGGUCCACCGUUACAGUUGGACACAGGCCAACUUCUCAAGAUUGCAAAACGUGGCGGCGUGACCGCAGUGGAGGCAGUGCAUGCAUGGCGCAAUGCACUGACGGGUGCCCCGCUCAACUUGACCCCCCAGCAGGUGGUGGCCAUCGCCAGCAAUGGCGGUGGCAAGCAGGCGCUGGAGACGGUCCAGCGGCUGUUGCCGGUGCUGUGCCAGGCCCACGGCUUGACCCCGGAGCAGGUGGUGGCCAUCGCCAGCAAUAUUGGUGGCAAGCAGGCGCUGGAGACGGUGCAGGCGCUGUUGCCGGUGCUGUGCCAGGCCCACGGCUUGACCCCCCAGCAGGUGGUGGCCAUCGCCAGCAAUAAUGGUGGCAAGCAGGCGCUGGAGACGGUCCAGCGGCUGUUGCCGGUGCUGUGCCAGGCCCACGGCUUGACCCCGGAGCAGGUGGUGGCCAUCGCCAGCCACGAUGGCGGCAAGCAGGCGCUGGAGACGGUCCAGCGGCUGUUGCCGGUGCUGUGCCAGGCCCACGGCUUGACCCCCCAGCAGGUGGUGGCCAUCGCCAGCAAUGGCGGUGGCAAGCAGGCGCUGGAGACGGUCCAGCGGCUGUUGCCGGUGCUGUGCCAGGCCCACGGCUUGACCCCCCAGCAGGUGGUGGCCAUCGCCAGCAAUAAUGGUGGCAAGCAGGCGCUGGAGACGGUCCAGCGGCUGUUGCCGGUGCUGUGCCAGGCCCACGGCUUGACCCCCCAGCAGGUGGUGGCCAUCGCCAGCAAUGGCGGUGGCAAGCAGGCGCUGGAGACGGUCCAGCGGCUGUUGCCGGUGCUGUGCCAGGCCCACGGCUUGACCCCCCAGCAGGUGGUGGCCAUCGCCAGCAAUAAUGGUGGCAAGCAGGCGCUGGAGACGGUCCAGCGGCUGUUGCCGGUGCUGUGCCAGGCCCACGGCUUGACCCCGGAGCAGGUGGUGGCCAUCGCCAGCCACGAUGGCGGCAAGCAGGCGCUGGAGACGGUCCAGCGGCUGUUGCCGGUGCUGUGCCAGGCCCACGGCUUGACCCCCCAGCAGGUGGUGGCCAUCGCCAGCAAUGGCGGUGGCAAGCAGGCGCUGGAGACGGUCCAGCGGCUGUUGCCGGUGCUGUGCCAGGCCCACGGCUUGACCCCGGAGCAGGUGGUGGCCAUCGCCAGCCACGAUGGCGGCAAGCAGGCGCUGGAGACGGUCCAGCGGCUGUUGCCGGUGCUGUGCCAGGCCCACGGCUUGACCCCCCAGCAGGUGGUGGCCAUCGCCAGCAAUAAUGGUGGCAAGCAGGCGCUGGAGACGGUCCAGCGGCUGUUGCCGGUGCUGUGCCAGGCCCACGGCUUGACCCCGGAGCAGGUGGUGGCCAUCGCCAGCCACGAUGGCGGCAAGCAGGCGCUGGAGACGGUCCAGCGGCUGUUGCCGGUGCUGUGCCAGGCCCACGGCUUGACCCCCCAGCAGGUGGUGGCCAUCGCCAGCAAUAAUGGUGGCAAGCAGGCGCUGGAGACGGUCCAGCGGCUGUUGCCGGUGCUGUGCCAGGCCCACGGCUUGACCCCGGAGCAGGUGGUGGCCAUCGCCAGCCACGAUGGCGGCAAGCAGGCGCUGGAGACGGUCCAGCGGCUGUUGCCGGUGCUGUGCCAGGCCCACGGCUUGACCCCUCAGCAGGUGGUGGCCAUCGCCAGCAAUGGCGGCGGCAGGCCGGCGCUGGAGAGCAUUGUUGCCCAGUUAUCUCGCCCUGAUCCGGCGUUGGCCGCGUUGACCAACGACCACCUCGUCGCCUUGGCCUGCCUCGGCGGGCGUCCUGCGCUGGAUGCAGUGAAAAAGGGAUUGGGGGAUCCUAUCAGCCGUUCCCAGCUGGUGAAGUCCGAGCUGGAGGAGAAGAAAUCCGAGUUGAGGCACAAGCUGAAGUACGUGCCCCACGAGUACAUCGAGCUGAUCGAGAUCGCCCGGAACAGCACCCAGGACCGUAUCCUGGAGAUGAAGGUGAUGGAGUUCUUCAUGAAGGUGUACGGCUACAGGGGCAAGCACCUGGGCGGCUCCAGGAAGCCCGACGGCGCCAUCUACACCGUGGGCUCCCCCAUCGACUACGGCGUGAUCGUGGACACCAAGGCCUACUCCGGCGGCUACAACCUGCCCAUCGGCCAGGCCGACGAAAUGCAGAGGUACGUGGAGGAGAACCAGACCAGGAACAAGCACAUCAACCCCAACGAGUGGUGGAAGGUGUACCCCUCCAGCGUGACCGAGUUCAAGUUCCUGUUCGUGUCCGGCCACUUCAAGGGCAACUACAAGGCCCAGCUGACCAGGCUGAACCACAUCACCAACUGCAACGGCGCCGUGCUGUCCGUGGAGGAGCUCCUGAUCGGCGGCGAGAUGAUCAAGGCCGGCACCCUGACCCUGGAGGAGGUGAGGAGGAAGUUCAACAACGGCGAGAUCAACUUCGCGGCCGACUGAUAACUCGAGGCUGCCUUCUGCGGGGCUUGCCUUCUGGCCAUGCCCUUCUUCUCUCCCUUGCACCUGUACCUCUUGGUCUUUGAAUAAAGCCUGAGUAGGAAGGUCGAGGCGGCCAACAACAAAAAAAAAAAAAAAAAAAAAAAAAAAAAAAAAAAAAAAAAAAAAAAAAAAAAAAAAAAAAAAAAAAAAAAAAAAAAAAAAAAAAAAAAAAAAAAAAAAAAAAAAAAAAAAAAAAAAAAAAAAAA |
| B2M TALEN RIGHT arm | AUGGGCGAUCCUAAAAAGAAACGUAAGGUCAUCGAUAUCGCCGAUCUACGCACGCUCGGCUACAGCCAGCAGCAACAGGAGAAGAUCAAACCGAAGGUUCGUUCGACAGUGGCGCAGCACCACGAGGCACUGGUCGGCCACGGGUUUACACACGCGCACAUCGUUGCGUUAAGCCAACACCCGGCAGCGUUAGGGACCGUCGCUGUCAAGUAUCAGGACAUGAUCGCAGCGUUGCCAGAGGCGACACACGAAGCGAUCGUUGGCGUCGGCAAACAGUGGUCCGGCGCACGCGCUCUGGAGGCCUUGCUCACGGUGGCGGGAGAGUUGAGAGGUCCACCGUUACAGUUGGACACAGGCCAACUUCUCAAGAUUGCAAAACGUGGCGGCGUGACCGCAGUGGAGGCAGUGCAUGCAUGGCGCAAUGCACUGACGGGUGCCCCGCUCAACUUGACCCCCCAGCAGGUGGUGGCCAUCGCCAGCAAUAAUGGUGGCAAGCAGGCGCUGGAGACGGUCCAGCGGCUGUUGCCGGUGCUGUGCCAGGCCCACGGCUUGACCCCCCAGCAGGUGGUGGCCAUCGCCAGCAAUAAUGGUGGCAAGCAGGCGCUGGAGACGGUCCAGCGGCUGUUGCCGGUGCUGUGCCAGGCCCACGGCUUGACCCCGGAGCAGGUGGUGGCCAUCGCCAGCAAUAUUGGUGGCAAGCAGGCGCUGGAGACGGUGCAGGCGCUGUUGCCGGUGCUGUGCCAGGCCCACGGCUUGACCCCCCAGCAGGUGGUGGCCAUCGCCAGCAAUGGCGGUGGCAAGCAGGCGCUGGAGACGGUCCAGCGGCUGUUGCCGGUGCUGUGCCAGGCCCACGGCUUGACCCCGGAGCAGGUGGUGGCCAUCGCCAGCAAUAUUGGUGGCAAGCAGGCGCUGGAGACGGUGCAGGCGCUGUUGCCGGUGCUGUGCCAGGCCCACGGCUUGACCCCCCAGCAGGUGGUGGCCAUCGCCAGCAAUAAUGGUGGCAAGCAGGCGCUGGAGACGGUCCAGCGGCUGUUGCCGGUGCUGUGCCAGGCCCACGGCUUGACCCCGGAGCAGGUGGUGGCCAUCGCCAGCCACGAUGGCGGCAAGCAGGCGCUGGAGACGGUCCAGCGGCUGUUGCCGGUGCUGUGCCAGGCCCACGGCUUGACCCCGGAGCAGGUGGUGGCCAUCGCCAGCCACGAUGGCGGCAAGCAGGCGCUGGAGACGGUCCAGCGGCUGUUGCCGGUGCUGUGCCAGGCCCACGGCUUGACCCCCCAGCAGGUGGUGGCCAUCGCCAGCAAUGGCGGUGGCAAGCAGGCGCUGGAGACGGUCCAGCGGCUGUUGCCGGUGCUGUGCCAGGCCCACGGCUUGACCCCGGAGCAGGUGGUGGCCAUCGCCAGCCACGAUGGCGGCAAGCAGGCGCUGGAGACGGUCCAGCGGCUGUUGCCGGUGCUGUGCCAGGCCCACGGCUUGACCCCGGAGCAGGUGGUGGCCAUCGCCAGCCACGAUGGCGGCAAGCAGGCGCUGGAGACGGUCCAGCGGCUGUUGCCGGUGCUGUGCCAGGCCCACGGCUUGACCCCGGAGCAGGUGGUGGCCAUCGCCAGCAAUAUUGGUGGCAAGCAGGCGCUGGAGACGGUGCAGGCGCUGUUGCCGGUGCUGUGCCAGGCCCACGGCUUGACCCCCCAGCAGGUGGUGGCCAUCGCCAGCAAUAAUGGUGGCAAGCAGGCGCUGGAGACGGUCCAGCGGCUGUUGCCGGUGCUGUGCCAGGCCCACGGCUUGACCCCCCAGCAGGUGGUGGCCAUCGCCAGCAAUAAUGGUGGCAAGCAGGCGCUGGAGACGGUCCAGCGGCUGUUGCCGGUGCUGUGCCAGGCCCACGGCUUGACCCCGGAGCAGGUGGUGGCCAUCGCCAGCCACGAUGGCGGCAAGCAGGCGCUGGAGACGGUCCAGCGGCUGUUGCCGGUGCUGUGCCAGGCCCACGGCUUGACCCCUCAGCAGGUGGUGGCCAUCGCCAGCAAUGGCGGCGGCAGGCCGGCGCUGGAGAGCAUUGUUGCCCAGUUAUCUCGCCCUGAUCCGGCGUUGGCCGCGUUGACCAACGACCACCUCGUCGCCUUGGCCUGCCUCGGCGGGCGUCCUGCGCUGGAUGCAGUGAAAAAGGGAUUGGGGGAUCCUAUCAGCCGUUCCCAGCUGGUGAAGUCCGAGCUGGAGGAGAAGAAAUCCGAGUUGAGGCACAAGCUGAAGUACGUGCCCCACGAGUACAUCGAGCUGAUCGAGAUCGCCCGGAACAGCACCCAGGACCGUAUCCUGGAGAUGAAGGUGAUGGAGUUCUUCAUGAAGGUGUACGGCUACAGGGGCAAGCACCUGGGCGGCUCCAGGAAGCCCGACGGCGCCAUCUACACCGUGGGCUCCCCCAUCGACUACGGCGUGAUCGUGGACACCAAGGCCUACUCCGGCGGCUACAACCUGCCCAUCGGCCAGGCCGACGAAAUGCAGAGGUACGUGGAGGAGAACCAGACCAGGAACAAGCACAUCAACCCCAACGAGUGGUGGAAGGUGUACCCCUCCAGCGUGACCGAGUUCAAGUUCCUGUUCGUGUCCGGCCACUUCAAGGGCAACUACAAGGCCCAGCUGACCAGGCUGAACCACAUCACCAACUGCAACGGCGCCGUGCUGUCCGUGGAGGAGCUCCUGAUCGGCGGCGAGAUGAUCAAGGCCGGCACCCUGACCCUGGAGGAGGUGAGGAGGAAGUUCAACAACGGCGAGAUCAACUUCGCGGCCGACUGAUAACUCGAGGCUGCCUUCUGCGGGGCUUGCCUUCUGGCCAUGCCCUUCUUCUCUCCCUUGCACCUGUACCUCUUGGUCUUUGAAUAAAGCCUGAGUAGGAAGGUCGAGGCGGCCAACAACAAAAAAAAAAAAAAAAAAAAAAAAAAAAAAAAAAAAAAAAAAAAAAAAAAAAAAAAAAAAAAAAAAAAAAAAAAAAAAAAAAAAAAAAAAAAAAAAAAAAAAAAAAAAAAAAAAAAAAAAAAAAA |
